# Supplementary material for: A lightweight intelligent compression method for fast Sea Level Anomaly data transmission
Source: PLoS One. 2025 Aug 18;20(8):e0327220. doi: 10.1371/journal.pone.0327220 (PMC12360586; doi:10.1371/journal.pone.0327220)
Supplement: Attachment — Submitted filename: comments.docx [file pone.0327220.s001.docx]

The article was reviewed under the title "A lightweight intelligent compression method for fast SLA data transmission".

Overall, the focus of the study is good. However, I would like to offer several recommendations that authors may find useful in the process of revising their manuscript:

1- For better understanding of the readers, it is better to explain Table 1 in more detail.
2- Table 4, which is intended for performance parameters, is empty and no data is listed in it.
3- Some sources are so old that newer sources need to be used for citation.
4- Section 3.1 states the following for the screening mechanism:” That is, if the number of null values in a single data grid exceeded 15%, the data were abandoned.”

What criteria is the threshold based on?
