# Supplementary material for: A lightweight intelligent compression method for fast Sea Level Anomaly data transmission
Source: PLoS One. 2025 Aug 18;20(8):e0327220. doi: 10.1371/journal.pone.0327220 (PMC12360586; doi:10.1371/journal.pone.0327220)
Supplement: Attachment — Submitted filename: Response Letter to Reviewer 2 _v2.docx [file pone.0327220.s002.docx]

***Response To Reviewer #2:***

Thank you for your diligent and professional review of our manuscript. We truly appreciate your expertise in the field of oceanography and your insightful comments, which have greatly contributed to the improvement of our paper.

We have carefully examined each of your suggestions and have made the necessary revisions accordingly. To facilitate the revision process, we have submitted a revised version of the manuscript with tracked changes.

Once again, we are grateful for your thorough evaluation and helpful feedback. Your expertise has been invaluable to us, and we sincerely thank you for your time and effort in reviewing our manuscript. The revisions will be presented in the article as comments in review mode, with a summary of the changes listed below:

*Q1: For better understanding of the readers, it is better to explain Table 1 in more detail.*

**Response: Thank you, and we accept your suggestion.**

The verification of detection and identification of mesoscale vortices has always been a partially subjective test, but the verification of the hybrid recognition algorithm mention.

*Q2: Table 4, which is intended for performance parameters, is empty and no data is listed in it.*

**Response: Thank you, and we accept your suggestion.**

Thank you for your suggestion. There was a problem with the verification work during the upload. We are sorry for the inconvenience caused to you, other reviewers and editors in reading. We will make supplements in the revised manuscript.

*Q3: Some sources are so old that newer sources need to be used for citation.*

**Response: Thank you, and we accept your suggestion.**

Thank you for your suggestion. We will supplement and cite some recently published literature related to the super-resolution improvement of Marine data compression in the revised manuscript.

*Q4: Section 3.1 states the following for the screening mechanism:” That is, if the number of null values in a single data grid exceeded 15%, the data were abandoned.”
What criteria is the threshold based on?*

**Response: Thank you, and we accept your suggestion.**

Thank you for your suggestion. Your suggestion is very incisive. Regarding this part of data processing and data availability, in this article, we have reduced the content and hidden some key explanations. In order to re-present them without increasing the volume of this article, We will quote the content about this part of data preprocessing from the articles previously published by our research group in the article paragraph you pointed out.
