# Supplementary material for: A lightweight intelligent compression method for fast Sea Level Anomaly data transmission
Source: PLoS One. 2025 Aug 18;20(8):e0327220. doi: 10.1371/journal.pone.0327220 (PMC12360586; doi:10.1371/journal.pone.0327220)
Supplement: Attachment — Submitted filename: comments_declet_1.docx [file pone.0327220.s003.docx]

The article titled " A lightweight intelligent compression method for fast SLA data transmission" was reviewed again.

This revision has significantly improved the manuscript. The authors have satisfactorily addressed my concerns.
